# Resolution enhancement in scanning electron microscopy using deep learning

**DOI:** 10.1038/s41598-019-48444-2

**Published:** 2019-08-19

**Authors:** Kevin de Haan, Zachary S. Ballard, Yair Rivenson, Yichen Wu, Aydogan Ozcan

**Affiliations:** 1 Electrical and Computer Engineering Department, University of California, Los Angeles, CA 90095 USA; 2 Bioengineering Department, University of California, Los Angeles, CA 90095 USA; 3 California NanoSystems Institute (CNSI), University of California, Los Angeles, CA 90095 USA; 4 Department of Surgery, David Geffen School of Medicine, University of California, Los Angeles, CA 90095 USA

**Keywords:** Imaging techniques, Nanoscience and technology

## Abstract

We report resolution enhancement in scanning electron microscopy (SEM) images using a generative adversarial network. We demonstrate the veracity of this deep learning-based super-resolution technique by inferring unresolved features in low-resolution SEM images and comparing them with the accurately co-registered high-resolution SEM images of the same samples. Through spatial frequency analysis, we also report that our method generates images with frequency spectra matching higher resolution SEM images of the same fields-of-view. By using this technique, higher resolution SEM images can be taken faster, while also reducing both electron charging and damage to the samples.

## Introduction

Scanning electron microscopy (SEM) is an important tool for characterization of materials at the nanoscale. By using electrons instead of photons for imaging samples, SEM can achieve sub-nanometer spatial resolution^1^, revealing topological and compositional features invisible to traditional light microscopy. Therefore, SEM is frequently employed in a wide range of fields such as material science, biomedicine, chemistry, physics, nanofabrication, and forensics, among others^2–4^. For example, new applications such as nanocutting, where a silicon wafer can be cut at m/s speeds using a diamond blade have been demonstrated^5–8^. Applications such as these require SEM characterization and therefore, as new tools to process or investigate the properties of silicon and other materials are developed, there will be an expanding need for improved electron microscopy tools.

However, when compared to light microscopy, the focused electron beam utilized by SEM is inherently more destructive to samples, especially soft and/or dielectric materials, resulting in electron charge build-up as well as deformation from absorption-based heating^9^. Consequently, these practical barriers prohibit many important samples such as biological specimens, polymers, and hydrogel-structures from being reliably characterized by SEM. There are, however, several approaches to mitigate the destructive effects of the electron beam. For example, it is common practice to coat the samples in e.g., gold, palladium, or iridium prior to imaging^10^. Additionally, shorter dwell times can be used during the electron beam scan to reduce the exposure to the sample. Though helpful, these approaches pose a performance trade-off: to reduce charging effects and sample deformation from heat one must alter the sample from its native state and/or incur increased noise in the acquired image^11^.

Although computational approaches for super resolution in electron microscopy have been previously demonstrated^12,13^, they require that a portion of the image be taken in high resolution or that the images have similar characteristics and contain sparse unique structures outside of a periodic topology. Other computational enhancements that have been applied to SEM images include denoising as well as deconvolution to reduce the spatial blur in the image caused by the finite beam size^14,15^. Alternative imaging techniques such as ptychography can also be used to increase the resolution in SEM, but these approaches require modification of the imaging set-up^16,17^.

Here, we present a deep learning-based approach to improve the lateral resolution of SEM images using a neural network. By training a convolutional neural network (CNN) with a set of co-registered high- and low-resolution SEM images of the same set of samples, we blindly super resolve individual SEM images, reducing sample charging and beam damage without losing image quality or adding extra sample preparation steps. In contrast to previous classical image enhancement methods, our approach can be implemented over a wide-range of sample types, and only requires a single SEM image as input. Furthermore, by using deep learning to perform super-resolution, the need for prior knowledge of the image and approximated models that estimate the forward imaging operator is eliminated. Super-resolution using a single input is also advantageous as it simplifies image acquisition and therefore improves the speed and broadens the number of possible applications. Additionally, by using a co-registered and experimentally acquired training image dataset, the network can partially account for possible aberrations and noise in the imaging system. This data-driven approach has the added benefit of reducing the scanning time of the electron beam, and thus increasing the imaging throughput by enabling the use of a lower magnification scan over a larger field-of-view without sacrificing image quality.

Deep neural networks have emerged as an effective method for statistical processing of images and have been shown to improve image quality and achieve super resolution of camera images^18^ and across several modalities of optical microscopy^19,20^. Once trained, the network can quickly process input SEM images in a feed-forward and non-iterative manner to blindly infer images with improved quality and resolution, thus making it an attractive and practical tool for rapid SEM image enhancement. Additionally, deep-learning based super-resolution has been proven to be more effective than other classical image enhancement techniques^18^.

## Imaging

We demonstrated the efficacy of our deep-learning based technique using a gold-on-carbon resolution test specimen [Ted Pella 617-a]. This test specimen has a random assortment of gold nanoparticles of varying sizes ranging from 5 nm to 150 nm immobilized on carbon, and is commonly employed to measure the resolution of SEM systems at different scales using the gaps between various gold nanoparticles. The network’s effectiveness when applied to a hydrogel coated with a thin layer of gold is also demonstrated.

The image dataset employed to train the CNN was made up of unique high- and low-resolution pairs of the test specimen, each taken from the same region of interest where there is a distribution of nanoparticles. The low-resolution images were taken at a magnification of 10000× (14.2 nm pixel size), while the high resolution images were taken at 20000× magnification (7.1 nm pixel size). We empirically found out that higher magnification ground truth images (>20000×) can lead to inaccurate inference results, and therefore limited the ratio of the label image magnification to the input image magnification as 2. The training SEM image pairs were taken by first capturing the high resolution images, and then lowering the magnification and imaging the same field of view. In both cases the image resolution is limited by the number of pixels and therefore the lower magnification images can be modeled as aliased versions of the higher resolution images. A Nova 600 DualBeam-SEM (FEI Company) was used with a 10 kV accelerating voltage, 0.54 nA beam current, and a monopole magnetic immersion lens for high resolution imaging. All images were acquired with 30 µs pixel dwell time.

For the hydrogel imaging experiments, the low-resolution images were taken at a magnification of 2500× (56.8 nm pixel size), and the corresponding high resolution images were taken at 10000× magnification and binned to give an effective magnification of 5000× (28.4 nm pixel size). These images were acquired using a 10 µs pixel dwell time.

## Co-registration

Once the high- and low-resolution image pairs were taken, they were co-registered before being inputted to the neural network for the training phase. These training images were first roughly matched to each other by cropping the center of each of the low-resolution images and using a Lanczos filter to up-sample the images. After this rough alignment, additional steps were taken to register the images with higher accuracy. First, image rotation and size misalignment were corrected by using the correlation between the two images to define an affine matrix which was then applied to the high resolution images. Next, local registration was performed using a pyramid elastic registration algorithm^21,22^. This algorithm breaks the images into iteratively smaller blocks, registering the local features within the blocks each time, achieving sub-pixel level agreement between the lower and higher resolution SEM images. The images were taken using automatic brightness and contrast adjustment. In order to account for possible fluctuations in the settings, both the high- and low-resolution images were normalized for use in the network training using the mean and standard deviation of the pixel values.

## Network Training

For the gold nanoparticles, 40 pairs of accurately registered images (924 × 780 pixels) were split into 1920 non-overlapping patches (128 × 128 pixels) which were then used to train the network. The hydrogel image dataset was made up of 131 pairs of 492 × 418 pixel images, which were cropped into 4542 overlapping 128 × 128 pixel patches. 372 of these patches were automatically removed from the training dataset using an experimentally determined correlation threshold due to beam damage creating severe dissimilarities between the images. The sizes of the training datasets were further increased by randomly rotating and flipping each image patch, and an identical network model was used for each dataset. The network model utilized in this work was a Generative Adversarial Network (GAN) which uses a generator network to create the enhanced images, and a discriminator network (D) that helps the generator network (G) to learn how to create realistic high-resolution images^23^. In addition to the standard discriminator loss, an L1 loss term was also added to ensure that the generated images are structurally close to the target, high-resolution images; the anisotropic total variation loss (TV) was also used to increase the sparsity of the output images and reduce noise. Based on this, the overall loss function for the generator network can be written as:

1 $${l}_{generator}={L}_{1}\{G(x),z\}+\alpha \times TV\{G(x)\}+\beta \times {[1-D(G(x))]}^{2}$$

where *x* is the low resolution input image to the generator network and *z* is the matching high resolution ground truth image corresponding to the same field-of-view. α and *β* are tunable parameters to account for the relative importance of the different loss terms. The L_1_ loss is the mean pixel difference between the generator’s output and the ground truth image, defined as^24^:

2 $${L}_{1}\{G(x),z\}=\frac{1}{M\times N}\sum _{i}\,\sum _{j}\,|{z}_{i,j}-G{(x)}_{i,j}|$$

where *i* and *j* are the pixel indices in an *M* × *N* pixel image. The anisotropic total variation loss is defined as^25^:

3 $$TV\{G(x)\}=\sum _{i}\,\sum _{j}\,(|G{(x)}_{i+1,j}-G{(x)}_{i,j}|+|G{(x)}_{i,j+1}-G{(x)}_{i,j}|)$$

The discriminator loss, on the other hand, penalizes the discriminator when it is unable to discriminate between the generated and the ground truth images, and is defined as^23^:

4 $${l}_{discriminator}=D{(G(x))}^{2}+{(1-D(z))}^{2}$$

**Figure 1:**
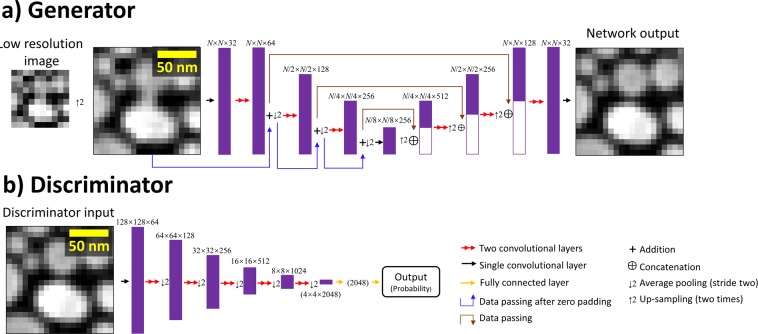
Diagram of the network structure. Every convolutional block is made up of two convolutional layers, each followed by a leaky rectified linear unit (ReLU) activation function. The second convolutional layer in each block changes the number of channels. (**a**) The structure of the generator portion of the network. (**b**) The structure of the discriminator portion of the network.

The discriminator loss, L1 loss, and the total variation loss make up 84%, 14%, and 2% of the total loss for the generator, respectively. The generator uses an adapted U-net structure^26^, while the discriminator uses a modified Visual Geometry Group (VGG) type network structure^27^. Details of these network architectures are shown in Fig. 1.

The network was implemented in Python (version 3.6.2) using the TensorFlow library (version 1.8.0). For both samples, the generator was trained for 48,000 iterations with the discriminator updating every fourth iteration to avoid overfitting. This took the network one hour and twenty minutes to train using a single Nvidia GTX 1080 Ti graphics processing unit (GPU) and an Intel Core i9-7900 processor. The same computer is able to infer 3.66 images per second, for an image size of 780 × 780 pixels. This inference time is 16 times faster than the low-resolution SEM imaging of the corresponding sample field-of-view; stated differently, real-time visualization of the super-resolved images, immediately after a low-resolution image is taken or while a new scan is ongoing, is feasible.

## Results

**Figure 2:**
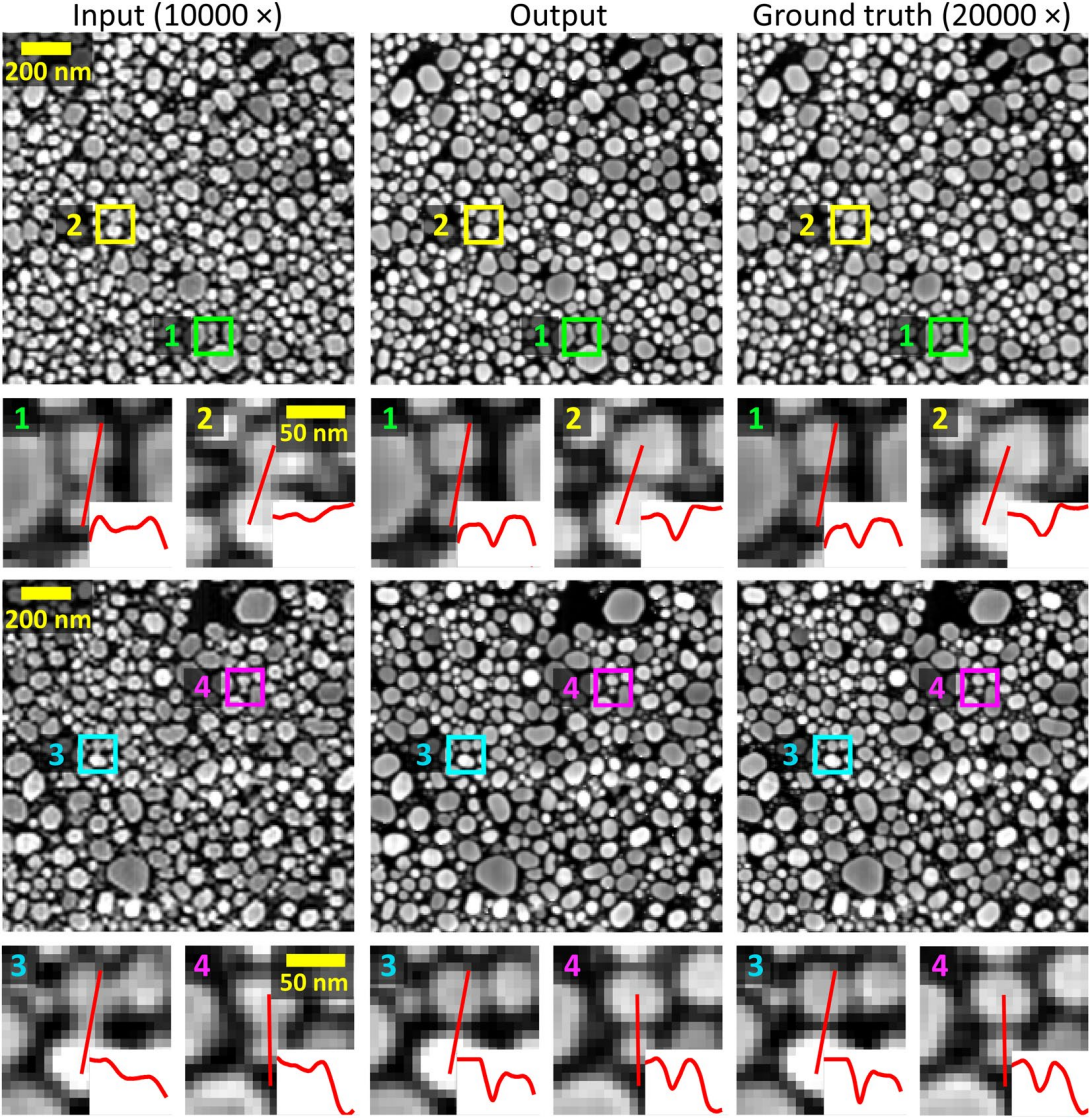
Examples of the up-sampled network input images compared to the output and ground truth SEM images. Cross sections of various spatial features with noticeable resolution enhancement are shown.

This super resolution technique allows us computationally to enhance the resolution of lower magnification SEM images such that the network’s output accurately matches the resolution given by the higher resolution SEM images of the same samples. A demonstration of this can be seen in Fig. 2, which reports several blindly tested examples of nanoparticles that are not clearly resolved in the input images, but become distinct after the application of the neural network. These fields of view are distinct from those used to train the network, but taken from different areas of the same sample. Pixel-intensity cross-sections are also reported to illustrate the resolution enhancement more clearly. From these examples we can see that the network is able to reveal spatial details that are not clear in the input (lower magnification) SEM images, matching at its output the corresponding higher magnification SEM images of the same fields-of-view. This is particularly evident in the gaps between the gold nanoparticles shown in Fig. 2.

**Figure 3:**
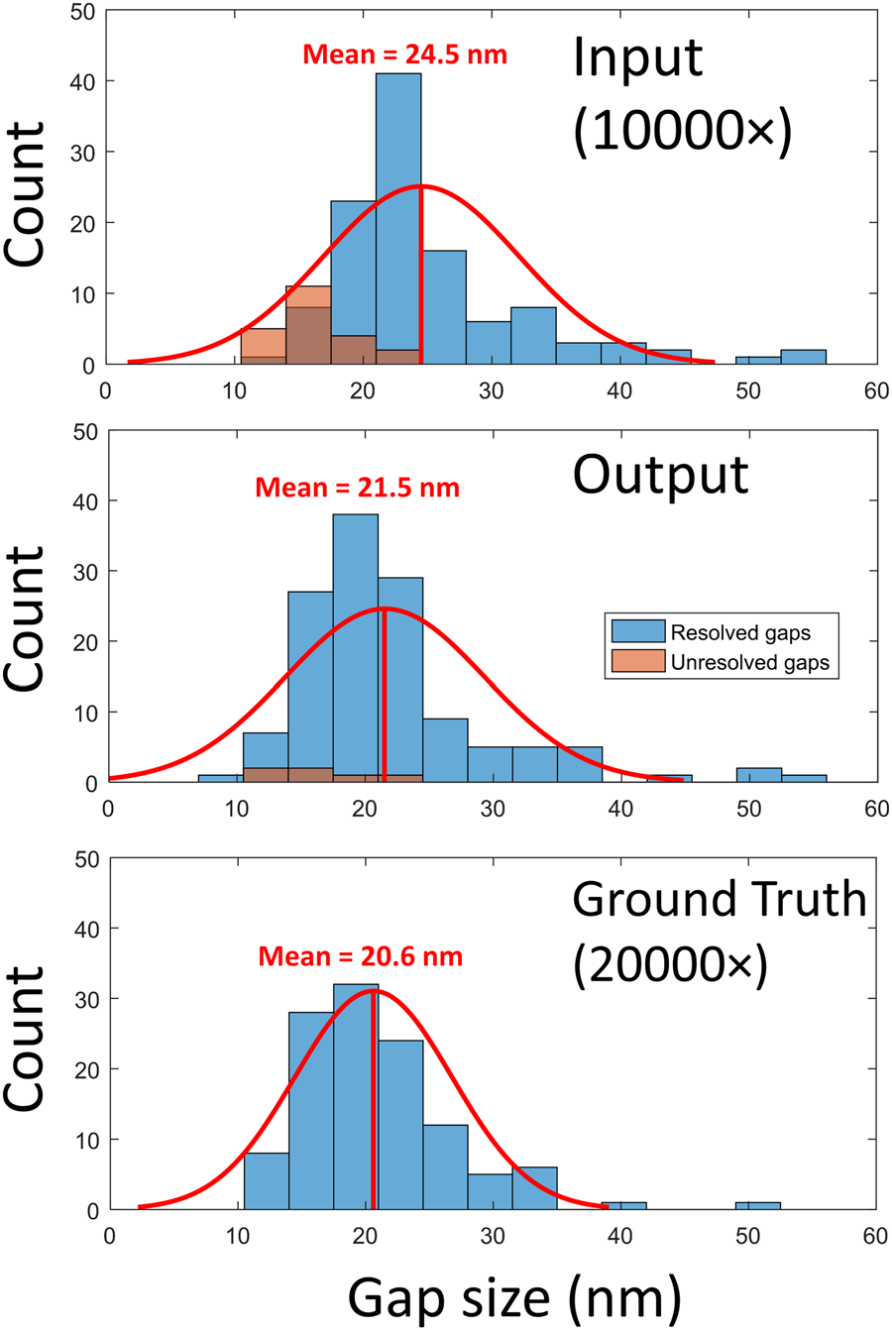
Histograms of the gap sizes inferred from the network input and the output images compared to the ground truth image. Total count changes among the histograms due to some of the gaps only being visible in specific images. In the input SEM images, 13.9% of the gaps were not detectible; the percentage of undetected gaps dropped to 3.7% for the output images. A Gaussian distribution, fitted to the gap histograms, with the corresponding mean gap size is also shown for each plot. The number of unresolved gaps in both the input and output images is also shown using a different color; unresolved gaps were not used for mean gap estimation. Pixel size per image is 7.1 nm; the input image is upsampled by a factor of 2.

In fact, Fig. 3 provides a statistical analysis of these gaps to quantify the enhancement provided by the trained network; for this analysis, 300 gaps between arbitrary adjacent nanoparticles were randomly selected using the high-resolution SEM images. They were then analyzed to determine whether the neighboring particles are resolvable, as well as to quantify the gap-size in the input image, output image, and target image. The gap width was defined as the distance between the points at which the intensity drops below 80% of highest intensity value of the adjacent particles, and a gap was determined to exist if the lowest intensity point between the particles fell below 60% of the peak value. In the input SEM image (lower magnification), 13.9% of these gaps were not detectible, i.e., could not be resolved (see Fig. 3). However, after super resolving the input SEM images using the trained network, the percentage of undetected gaps dropped to 3.7%. Additionally, the *average absolute difference* between the measured gap sizes in the low- and high-resolution SEM images decreases from 3.8 nm to 2.1 nm after passing through the network.

**Figure 4:**
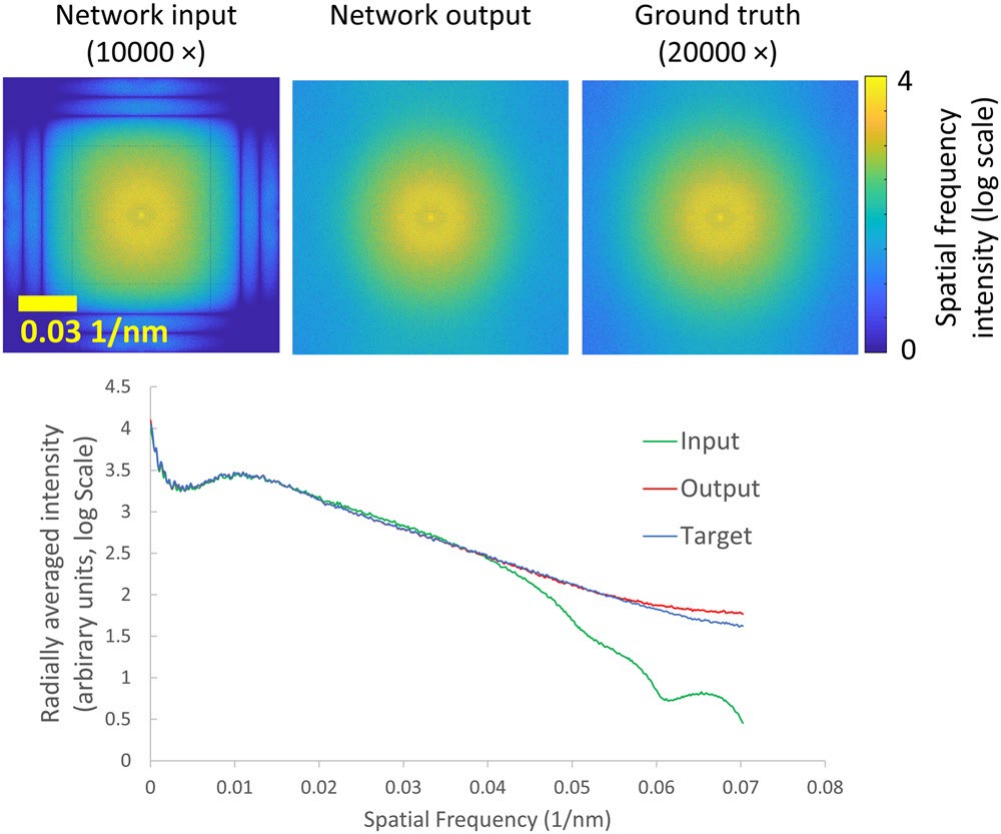
Top: spatial frequency distributions of the average of five up-sampled input, output, and ground truth images are compared. Bottom: radially-averaged plot of the above distributions. Analysis was performed on the uncropped versions of the SEM images shown in Fig. 2 as well as three additional images.

Another way to illustrate the resolution improvement is reported in the spatial frequency analysis shown in Fig. 4. This figure compares the magnitudes of the spatial frequencies for the low- and high-resolution SEM images as well as those of the network output images. From this comparative analysis we can see that the network enhances the high frequency details of the input SEM image such that the spatial frequency distribution of the network output image is consistent with the high-resolution SEM image – including the spatial frequencies that are aliased in the input image due to the large pixel size.

**Figure 5:**
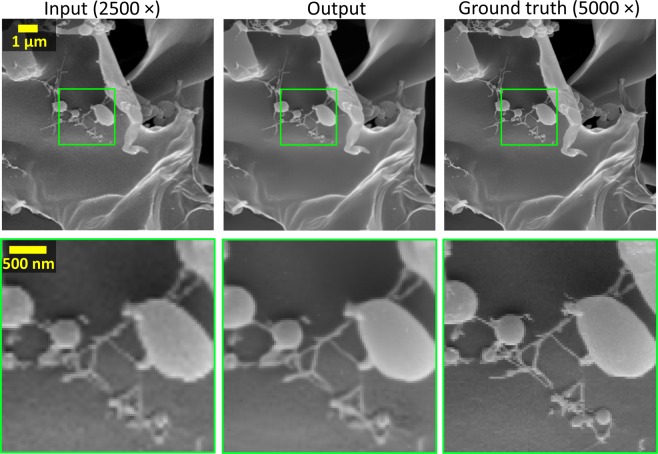
Examples of the up-sampled hydrogel network input images compared to the network output and the ground truth SEM images. Top: full field of view. Bottom: zoomed in region of interest. As the hydrogel sample is partially damaged during the imaging process, the ground truth images have a slightly different structure than the input images. This sample damage makes subpixel image co-registration impossible, which reduces the quality of the network training and limits the success of its inference, compared to earlier presented results.

To demonstrate the performance of the network when applied to a sample that is prone to beam damage, a hydrogel sample was used. In this case, the network was able to improve the image quality by sharpening and denoising it (Fig. 5). However, the electron beam-induced damage of the sample during imaging makes accurate co-registration impossible and therefore reduces the success of the network training process and its inference. An example of this can be seen in Fig. 5. In cases like this, the performance of the super-resolution network will decrease as the high- and low-resolution images used to train the network become more dissimilar due to movement of the specimen. However, once a network has been trained, the inference process requires only a single input image. Therefore, our approach can help to mitigate some of the sample deformation caused by the radiation as the low-resolution image can be captured using reduced beam intensity.

## Conclusions

Taken together, deep learning-based super resolution is shown to be a powerful and practical tool to computationally improve the resolution in SEM. The 2-fold increase in resolution demonstrated here allows for a *four-fold* reduction of the number of electrons which must interact with the sample to acquire an SEM image, in turn enabling a four-fold increase in the speed of image acquisition. While the demonstrated method is less effective when trained using samples that are prone to significant beam damage or other movement during the acquisition of the training dataset, it could benefit the characterization of samples that present limited charging or beam-induced damage by reducing the electron exposure without sacrificing the image quality. This would allow for higher resolution imaging of a variety of biological materials and nanofabricated samples that previously could not be characterized adequately by SEM. While we demonstrated the effectiveness of our network in the ideal case, where the training and testing datasets are made up of the same type of sample, deep learning-based super-resolution techniques have been shown to generalize to other types of samples or magnification factors, where larger datasets were used or there were strong similarities between different types of samples^16^; this can be further improved through transfer learning^15^, which can be performed in almost real time by acquiring a few calibration images corresponding to a new type of sample of interest.
